# Runx1 Loss Minimally Impacts Long-Term Hematopoietic Stem Cells

**DOI:** 10.1371/journal.pone.0028430

**Published:** 2011-12-01

**Authors:** Xiongwei Cai, Justin J. Gaudet, James K. Mangan, Michael J. Chen, Maria Elena De Obaldia, Zaw Oo, Patricia Ernst, Nancy A. Speck

**Affiliations:** 1 Abramson Family Cancer Research Institute and Department of Cell and Developmental Biology, University of Pennsylvania, Philadelphia, Pennsylvania, United States of America; 2 Department of Biochemistry, Dartmouth Medical School, Hanover, New Hampshire, United States of America; 3 Department of Genetics, Dartmouth Medical School, Hanover, New Hampshire, United States of America; Emory University, United States of America

## Abstract

*RUNX1* encodes a DNA binding subunit of the core-binding transcription factors and is frequently mutated in acute leukemia, therapy-related leukemia, myelodysplastic syndrome, and chronic myelomonocytic leukemia. Mutations in *RUNX1* are thought to confer upon hematopoietic stem cells (HSCs) a pre-leukemic state, but the fundamental properties of Runx1 deficient pre-leukemic HSCs are not well defined. Here we show that Runx1 deficiency decreases both apoptosis and proliferation, but only minimally impacts the frequency of long term repopulating HSCs (LT-HSCs). It has been variously reported that Runx1 loss increases LT-HSC numbers, decreases LT-HSC numbers, or causes age-related HSC exhaustion. We attempt to resolve these discrepancies by showing that Runx1 deficiency alters the expression of several key HSC markers, and that the number of functional LT-HSCs varies depending on the criteria used to score them. Finally, we identify genes and pathways, including the cell cycle and p53 pathways that are dysregulated in Runx1 deficient HSCs.

## Introduction

One of the most commonly mutated genes in leukemia is *RUNX1*, which encodes a DNA-binding subunit of the heterodimeric core-binding factors. Chromosomal translocations involving *RUNX1* are found in multiple hematopoietic malignancies including acute myelogenous leukemia (AML), acute lymphocytic leukemia (ALL), and therapy-related AML and myelodysplastic syndrome (MDS). For example, the t(8;21)(q22;q22) which fuses Runx1 (or AML1) to the ETO protein (encoded by *RUNX1T1*) is found in 5–12% of *de novo* AML. Mono- or biallelic deletions, missense, nonsense, and frameshift mutations in *RUNX1* are also found in patients with *de novo* AML, MDS, chronic myelomonocytic leukemia, and in therapy-related MDS and AML [1], [2], [3], [4], [5], [6], [7]. Missense mutations are most commonly found in the DNA binding Runt domain, with other mutations scattered throughout the coding sequences. *RUNX1* mutations are found in approximately 5–6% of de novo AML patients, but the mutation frequency is reportedly quite high in certain leukemia subtypes [7]. For example, a recent analysis of 449 *de novo* AML patients with normal karyotype or non complex chromosomal imbalances identified *RUNX1* mutations in 32.7% of cases, including 65% of the least differentiated French-American-British (FAB) subtype (AML M0) [6]. The mechanism by which Runx1 loss contributes to AML or MDS is not entirely clear, nor is it understood why *de novo* AML associated with biallelic loss of function *RUNX1* mutations confers a considerably worse prognosis than, for example, *de novo* AML with the (8;21) translocation [6], [8], [9].

Chromosomal translocations and mutations in *RUNX1* can be initiating events that occur in HSCs, after which leukemias clonally evolve through the acquisition of secondary mutations [6], [10], [11]. A thorough characterization of the cell-autonomous impact of Runx1 loss on HSCs and progenitors is therefore essential for understanding the pre-leukemic state conferred by *RUNX1* mutations, and for identifying potential therapeutic targets for eliminating leukemic or preleukemic HSCs. Germline deletion of *Runx1* in mice is lethal and blocks blood cell formation [12], [13]. However, if Runx1 function is lost or compromised after HSCs in the fetus have formed, lineage negative Sca1^+^ c-Kit^+^ (LSK) cells and committed myeloid progenitors are not lost, but instead expand several fold in the bone marrow [14], [15], [16], [17], [18]. Mice with Runx1 deficient HSCs do not spontaneously develop leukemia, but are sensitized to leukemia caused by experimentally induced secondary mutations [18]. It is not entirely clear which specific properties of Runx1 deficient HSCs contribute to the pre-leukemic state. Presumably, though, for leukemia to evolve, Runx1 deficient HSCs must self-renew and persist in the bone marrow, as shown to be the case for HSCs expressing the t(12;21) product, TEL-AML1 [10].

Here we analyzed the cell-autonomous properties of Runx1 deficient HSCs. Deletion of *Runx1* expanded the number of LSK cells, consistent with all previous reports [15], [16], [17], [18], [19]. All subpopulations of Runx1 deficient LSK cells displayed a G_1_ cell cycle delay and decreased apoptosis. The number of functional Runx1 deficient LT-HSCs in the young adult bone marrow was either moderately decreased or unchanged, depending on whether contribution to peripheral blood or bone marrow was assessed. Runx1 deficiency influenced the expression of several LT-HSC markers, which may explain some of the contradictory reports in the literature on the effect of Runx1 deletion on phenotypic LT-HSCs [18], [20]. Finally, we report on the genes deregulated upon Runx1 deletion, and the potential pathways that are affected.

## Methods

### Mice

Mice were housed in microisolator cages in a pathogen-free animal facility and were treated according to Dartmouth's and the University of Pennsylvania's Animal Resources Center and IACUC protocols. The colonies of *Runx1^f/f^*; *Mx1-Cre* and *Runx1^f/f^*;*Vav1-Cre* mice were generated and maintained as described previously [14], [15]. Genotyping for the Tg(*Mx1-Cre*) allele was performed by polymerase chain reaction (PCR) using 1 µl of 10 µM internal control forward primer (oIMR0042, 5′ CTA GGC CAC AGA ATT GAA AGA TCT 3′), 1 µl of 10 µM internal control reverse primer (oIMR0043, 5′ GTA GGT GGA AAT TCT AGC ATC ATC C 3′), 1 µl of 10 µM forward primer (oIMR1084, 5′ GCG GTC TGG CAG TAA AAA CTA TC 3′), 1 µl of 10 µM reverse primer (oIMR1085, 5′ GTG AAA CAG CAT TGC TGT CAC TT 3′). Genotyping for the *Runx1^f^* allele (*Runx1^tm3Spe^*), Tg(*Vav1-Cre*) [21], and evaluation of excision were described previously [14], [15].

### Transplant studies

F1 progeny of either 129S1/SvImJ (Ly5.2) females crossed to B6.SJL-*Ptprc^a^ Pepc^b^/*BoyJ (Ly5.1) males (JAX, Bar Harbor, ME), or C57BL6/J (Ly5.2) females crossed to B6.SJL-*Ptprc^a^ Pepc^b^/*BoyJ males were used as recipients for *Runx1^f/f^*;*Mx1-Cre, Runx1^f/f^*;*Vav1-Cre,* and *Runx1^f/f^* cells. All donor cells were Ly5.2 (CD45.2^+^). Bone marrow chimeras were established by injecting donor fetal or bone marrow cells along with 2×10^5^ B6.SJL (carrier CD45.1/Ly5.1) marrow cells into lethally-irradiated (split dose 900–1200 cGy 3 hours apart) recipients. Engraftment was scored as ≥1% donor derived cells.

Serial transplants were initiated by injection of different doses of donor bone marrow cells into irradiated B6.SJL-*Ptprc^a^ Pepc^b^/*BoyJ recipients, without competitors. Reconstitution was assessed two months later and 2×10^6^ bone marrow cells from each primary recipient were transplanted into four secondary B6.SJL-*Ptprc^a^ Pepc^b^/*BoyJ recipients. 2×10^6^ bone marrow cells from each secondary recipient were transplanted into one or two tertiary recipients, and from each reconstituted tertiary recipient into a fourth recipient.

### Induction of Tg(*Mx1-Cre*) and deletion of *Runx1^f^*

Stable donor (*Runx1^f/f^*; *Mx1-Cre;* or *Runx1^f/f^*) reconstitution of recipient hematopoiesis was confirmed beginning at one month post transplant by multicolor flow cytometry. Recipient mice each received 3 intraperitoneal injections of pIpC (GE Healthcare, Piscataway NJ) [weight of mouse (g)×10 + 50]  =  µl of 1 mg/ml pIpC]. Deletion of *Runx1^f^* at a gross level was determined by PCR of marrow or peripheral blood cells [15] and clonally by PCR of individual colonies derived from CFU-C cultures. The latter indicated that deletions were >99% efficient (not shown).

### Cell purification and flow cytometry

Flow cytometry analyses were performed with a FACSCalibur, FACSAria, or LSRII (BD Bioscience, San Jose CA) equipped with CellQuest Pro or FACS Diva acquisition software. Subsequent data analyses were carried out using Flowjo (Treestar Inc, Ashland OR). Lineage depletion of bone marrow was achieved using the MACS Lineage Cell Depletion Kit, MidiMACS LS Separation units, and the QuadroMACS Multistand (Miltenyi) according to the manufacturer's instructions. In some cases erythrocytes were lysed in an RBC Lysis Buffer (eBioscience, San Diego, CA) prior to lineage depletion. A Cellometer (Nexcelom Bioscience, Lawrence MA) was used to count nucleated cells. The following monoclonal antibodies (BD Bioscience and/or eBioscience, San Diego CA) were used for staining: FITC/PE-Cy7-Sca1 (E13-161.7, D7), APC/APC-Cy7-cKit (2B8), PE/PE-Cy5-CD135 (A2F10), PE-Cy5/APC-CD45.1 (A20), FITC/APC-CD45.2 (104), FITC/PE-CD48 (BCM1), CD34 (RAM34), Mac1 (M1/70), Gr1 (RB6-8C5), CD3e (17A2), B220 RA3-6B2), Ter119 (TER119), and eFluor 450 or PE-Cy5 Streptavidin. Biotin-CD150 (TC15 12F12.2) was from BioLegend (San Diego CA). Dead cells were stained with Propidium Iodide (PI) (Sigma, St. Louis MO), 7-AAD, or ToPro-3 (Molecular Probes/Invitrogen, Carlsbad CA).

### Cell cycle analyses

HSCs were sorted into 100% fetal bovine serum (FBS) at 4°C using a FACSAria cell sorter. Sorted cells were centrifuged for 5 minutes at 300 x g, the supernatant removed, and pelleted cells resuspended in 1x HBSS containing 1 g/L Glucose, 20 mM HEPES, and 50 µg/ml Verapamil (Molecular Probes/Invitrogen). Hoechst 33342 (Molecular Probes/Invitrogen) was added to 10 µg/ml, and cells were incubated at 37°C for 45 minutes. Pyronin Y (Sigma) was added to 1 µg/ml and cells incubated for a further 15 minutes at 37°C. Cells were washed 1X, and immediately analyzed with the FACSAria.

Mice received BrdU intraperitoneally (1 mg/6 g) followed by administration in drinking water (1 mg/mL) for 3 days. Bone marrow cells were stained and analyzed according to the manufacturer's instructions (BD Bioscience).

### Gene expression analyses

Cell populations were sorted under sterile conditions at 4°C directly into an RNA lysis buffer, RLT containing β-mercaptoethanol (Qiagen, Valencia CA) and lysed by incubation at 42°C for 1 hour followed by 1 minute of vortexing. Total RNA was DNaseI-treated on Rneasy columns, and purified according to the manufacturer's instructions (Qiagen) except that the RNase-free water used to elute total RNA was heated to 70°C before applying to the columns. Total RNA quantity and integrity were verified using an Agilent Bioanalyzer and Pico kit (Agilent Technologies, Santa Clara CA). First and second round cDNA synthesis, and the first amplification reaction, was performed using the RiboAmp Kit (Arcturus, Foster City CA). 450 ng of cDNA was then used as template for a second *in vitro* transcription in which biotin-labeled aRNA was generated with the BioArray High Yield RNA Transcript labeling kit (T7) (Enzo Life Sciences, Plymouth Meeting PA) according to the manufacturer's instructions. Biotinylated aRNA was submitted to the Shared Resource Microarray Facility at Dartmouth Medical School for fragmentation and hybridization to Affymetrix Mouse 430 2.0 GeneChip arrays (Affymetrix, Santa Clara CA). Affymetrix CEL files were uploaded using the Microsoft Excel Add-in BRB Array Tools v3.7.0 and data normalized with the Robust Multichip Average (RMA) algorithm. A class comparison was performed using the random variance model, with a significance-threshold of 0.005 to identify differentially expressed probesets. Data were filtered to exclude any probeset having a mean fold change of less than 1.5 between classes, and non-annotated probesets were eliminated from further analyses. Microarray data were analyzed by gene set enrichment analysis (GSEA) [22] according to the online user guide. Venn diagrams were generated using VENNY [23].

RNA for qRT-PCR was purified by miRNeasy Mini Kit (Qiagen), and total RNA reverse transcribed (Applied Biosystems, Carlsbad CA). Reaction mixtures for quantitative PCR contained 5 nM of template DNA and 50 µM of primer DNA in 50 mM NaCl and 1 mM Mg^++^. A three step PCR was performed for 35 cycles. Denaturation was at 94°C for 20 seconds, annealing at 55°C for 20 seconds, and extension at 72°C for 30 seconds. Primers and probes are listed in Table 1.

**Table 1:** Primers for qRT-PCR.

Gene	Forward Primer	Reverse Primer	Taqman probe
*Cd34*	AGGCTCTGGAACTCCACACACTTT	TAA GCA TAT GGC TCG GTG GGT GAT	
*Cd48*	TGAGAGTGCTGCGTGAAACTGAGA	ATGCTGGTCCTTTACCTCACACGA	
*Cd48*	ATCGTGTGAGGTAAAGGACCAGCA	AGGATTGCTGACTTG GCAGGTGTA	
*Gadph*	CATGGCCTTCCGTGTTCCTA	TGTCATCATACTTGGCAGGTTTCT	CCCAATGTGTCCGTCGTGGATCTGA
*Hprt*	CTCCTCAGACCGCTTTTTGC	TAACCTGGTTCATCATCGCTAATC	
*Mpl*	TATTGGCAGCAGCCCTGAA	TGG ATG GTG TTG AGG ATG GAT A	
*Ndn*	CGTCCAGCAGAATTACCTGAAGT	CATGATCTGCATCTTGGTGATTTC	CCAGCGTGTGCCCCACATCG
*Nov*	GAAGCATATTGGTTGAGGCAAAT	GTGGGACAACTTCATTATGTTTCCT	TTGCTGCGGCATGGCCCA
*Prdm16*	TCATCCCAGGAGAGCTGCATCAAA	ATCACAGGAACACGCTACACGGAT	
*Slamf1*	AAACCCAGGAGAACGAGAATG GGA	GTT GCT TGC GGT GCA GTT GTA GAT	
*Tp53*	AAAGGATGCCCATGCTACAGAGGA	AGTAGACTGGCCCTTCTTGGTCTT	

### Administration of 5-fluorouracil

Mice were injected 5-fluorouracil (150 mg/kg) (Sigma) on days 0, 7, and 14, and both blood and bone marrow analyzed by flow cytometry.

## Results

### Runx1 regulates LSK pool size by a cell autonomous mechanism

**Figure 1:**
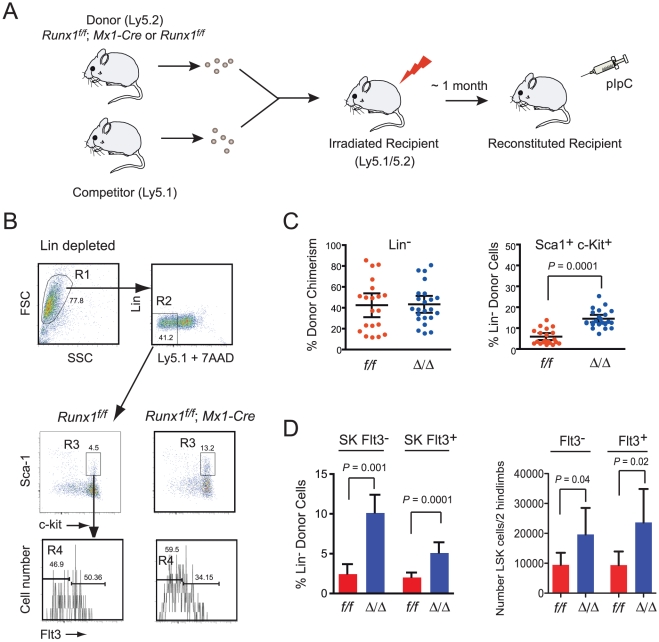
Runx1 deletion expands the phenotypic hematopoietic stem/progenitor pool in a cell-autonomous manner. A. Schematic diagram of the experimental strategy. Mice stably engrafted with donor and competitor cells were injected with pIpC, and sacrificed for analyses. B. Representative FACS analysis of lineage depleted donor-derived bone marrow LSK cells and Flt3^-^ and Flt3^+^ fractions thereof 5 weeks post pIpC injection. C. Donor chimerism within the Lin^-^ fraction of recipient marrow. Upon deletion of *Runx1* (∶/∶), there was no change in the percent donor contribution to the Lin^-^ fraction of cells (left panel), but within the donor-derived Lin^-^ fraction there was a significant increase in the percentage of Sca1^+^c-Kit^+^ cells (right panel). Dots show the distribution of donor chimerism, the horizontal lines reflect the mean (±95% CI). In animals whose bone marrow contained <30% Runx1-deficient Lin^-^ cells the difference was also significant (*P*<0.0001, not shown). D. Contribution of enriched donor LT-HSC/ST-HSCs (LSK Flt3^-^) and MPP/LMPPs (LSK Flt3^+^) to recipient bone marrow (mean ± 95% CI). Left panel shows percent contribution, *f/f* n = 10, ∶/∶ n = 12. Right panel shows total numbers, *f/f* n = 14, ∶/∶ n = 18.

We determined if the expansion of LSK cells that occurs upon conditional deletion of *Runx1* in adult bone marrow reflects a true cell-autonomous role of Runx1 in regulating LSK pool size. We transplanted total bone marrow from *Runx1^f/f^; Mx1-Cre* or *Runx1^f/f^* donor mice (Ly5.2) together with wild type competitor (Ly5.1) bone marrow into lethally irradiated congenic recipients (Ly5.1/5.2) (Figure 1A). After confirming hematopoietic reconstitution, we deleted Runx1 and analyzed Ly5.2^+^ donor-derived cells at 5 weeks post deletion. Consistent with all previous reports [15], [16], [18], [19], [20] we observed an expansion of Sca-1^+^ c-kit^+^ cells within the Lin^-^ population (Figure 1B,C). This expansion involved both LSK Flt3^-^ cells (LSKF^-^), which include all long term and short term repopulating HSCs (LT-HSCs and ST-HSCs), and LSKF^+^ cells which consist of early multipotent progenitors (MPPs) and lymphoid-primed MPPs (LMPPs) (Figure 1B,D) [24], [25]. The increase in LSK cells was significant regardless of the extent to which the bone marrow was reconstituted with donor-derived cells, even at low donor∶competitor ratios (not shown), indicating that expansion was not secondary to the lymphopenia or thrombocytopenia that result from Runx1 deficiency [15], [16]. Because the deletion was performed in the context of a bone marrow chimera, the expansion was not caused by deletion in the bone marrow niche, nor from enhanced engraftment. We conclude that Runx1 has a specific, cell autonomous role in regulating homeostatic LSK cell numbers.

### Effects of Runx1 loss on LT-HSCs

**Figure 2:**
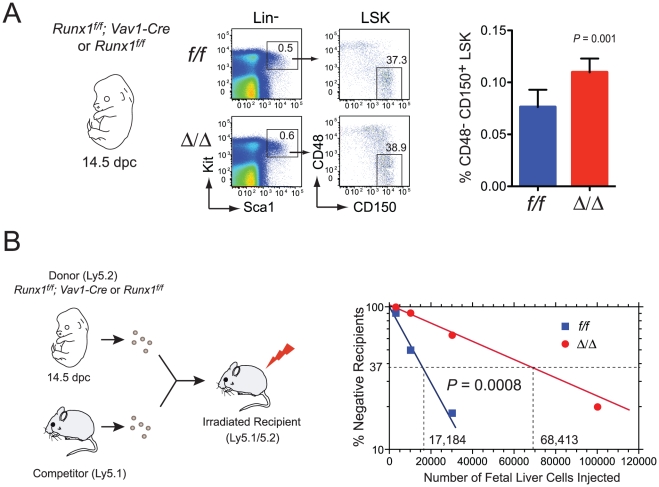
Effect of Runx1 deletion on fetal HSCs. A. The percentage of phenotypic Runx1 deficient fetal liver HSCs (14.5 dpc) defined using SLAM markers. Runx1 was deleted in fetal HSCs using Vav1-Cre. The number of fetal liver cells was equivalent. Plots indicate the percentage of CD48^-^ CD150^+^ LSK cells in the fetal liver; error bars represent 95% CI. Data are compiled from 7 *Runx1^f/f^* (*f/f*) and 6 *Runx1^f/f^; Vav1-Cre* fetuses (Δ/Δ) in two experiments. B. Competitive limit dilution transplant. Dilutions of 14.5 dpc fetal liver cells (Ly5.2^+^) were injected along with 2×10^5^ Ly5.1^+^ adult bone marrow cells into irradiated Ly5.1/5.2 recipients. The contribution of Ly5.2^+^ cells to peripheral blood was assessed, and recipients with ≥5% donor-derived cells at ≥16 weeks were deemed reconstituted. Data are compiled from three different experiments with a total of 6 to 13 mice per data point. The frequency of LT-HSCs in the wild type fetal was 1 in 17,184 (95% CI range  =  1 in 22,355 to 1 in 13,208). The frequency of LT-HSCs in the *Runx1^f/f^;Vav1-Cre* fetal liver was 1 in 68,413 (range  =  1 in 93,523 to 1 in 50,045).

**Figure 3:**
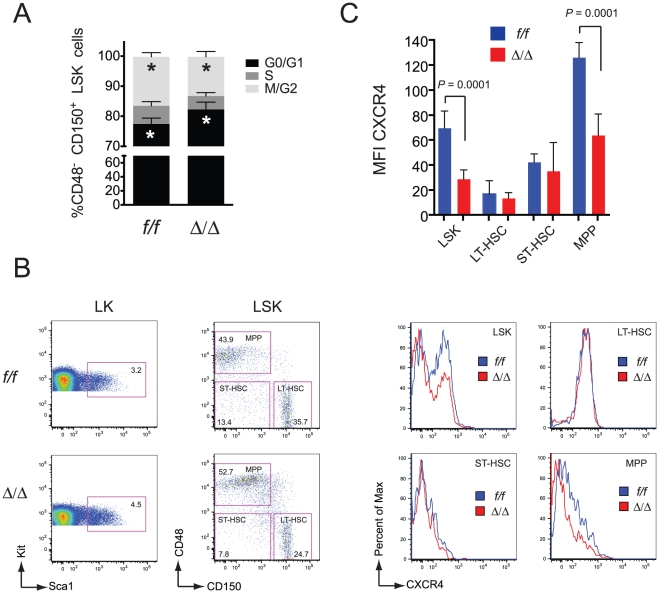
Proliferation profile and CXCR4 expression on Runx1 deficient fetal liver HSCs. A. Cell cycle analysis of sorted CD48^-^CD150^+^ LSK cells stained with Hoechst and analyzed by FACS. Error bars represent 95% CI. Differences between *f/f* and ∶/∶ cells in G_0_/G_1_ and M/G_2_ were significant (asterisks, *P* = 0.003 and 0.004, respectively). n = 6. B. Representative scatter plots for analysis of LSK and phenotypic LT-HSC, ST-HSC, and MPP populations are shown on left, and histograms for CXCR4 levels on the right. CD48^-^ CD150^-^ LSK cells are labeled ST-HSC based on analysis by Foudi et al. in adult marrow [67]. C. Analysis of CXCR4 levels on Runx1 deficient fetal liver HSCs. The mean fluorescence intensity (MFI) of CXCR4 is plotted for LSK, phenotypic LT-HSC (CD48^-^CD150^+^ LSK), ST-HSC (CD48^-^CD150^-^ LSK), and MPP (CD48^+^CD150^-^ LSK) populations (n = 5 or 6 fetuses, error bars represent 95% CI). Significance was determined by unpaired, two-tailed Student's t-test.

Since LSK expansion was not affected by the extent of chimerism, we switched to a simpler deletion strategy for many of the subsequent experiments, using Vav1-Cre. Vav1-Cre deletion, which is restricted to blood cells, initiates at 11.5 days post coitus (dpc) in the fetus, and is approximately ∼95% efficient by 13.5 dpc in fetal liver cells [14], [21]. We examined the phenotypic and functional consequences of Runx1 loss in two different LT-HSC populations in *Runx1^f/f^;Vav1-Cre* mice; from the 14.5 dpc fetal liver, and from the bone marrow of young (6–8 week old) adults. The percentage of phenotypic LT-HSCs in the 14.5 dpc fetal liver, as determined using SLAM markers [26], was 1.4 fold higher in *Runx1^f/f^;Vav1-Cre* fetuses than in *Runx1^f/f^* fetuses (Figure 2A). However, a competitive limit dilution transplant demonstrated that *Runx1^f/f^;Vav1-Cre* fetal livers had 4-fold fewer HSCs when the percentage of total donor-derived cells in peripheral blood was assessed, suggesting that LT-HSCs were functionally impaired (Figure 2B). We examined several potential underlying causes of the moderately impaired engraftment. All 14.5 dpc fetal liver HSCs capable of engraftment are in the G_1_ phase of the cell cycle [27], so we determined whether Runx1 deficient HSCs had fewer cells in G_1_ by Pyronin Y/Hoechst staining. We found, on the contrary, that *Runx1^f/f^;Vav1-Cre* fetal livers had significantly more CD48^-^ CD150^+^ LSK cells in G_1_ as compared to *Runx1^f/f^* mice (Figure 3A). Since essentially all fetal liver LT-HSCs are proliferating [27] the accumulation of cells in G_1_ reflects a delay in cell cycle progression, consistent with many previous reports that Runx1 promotes the G_1_ to S transition (reviewed in [28], [29], [30], [31], [32], [33]). Although one might predict that an HSC population with a greater proportion of G_1_ phase cells would provide superior engraftment, their lower proliferation rate might confer a competitive disadvantage. Jacob *et al.* [18] reported that chemokine receptor 4 (CXCR4) levels were decreased on adult Runx1 deficient LSK cells, and homing of carboxyfluorescein succinimidyl ester (CFSE)-labeled total bone marrow was lower. We also observed decreased levels of CXCR4 on Runx1 deficient fetal liver LSK cells, but this was confined to the phenotypic MPP population (CD48^+^ CD150^-^ LSK), and was not apparent on LT-HSCs or ST-HSCs (Figure 3B,C). No differences in the percentage of early apoptotic (7AAD^-^ Annexin V^+^) HSC populations were observed (not shown).

**Figure 4:**
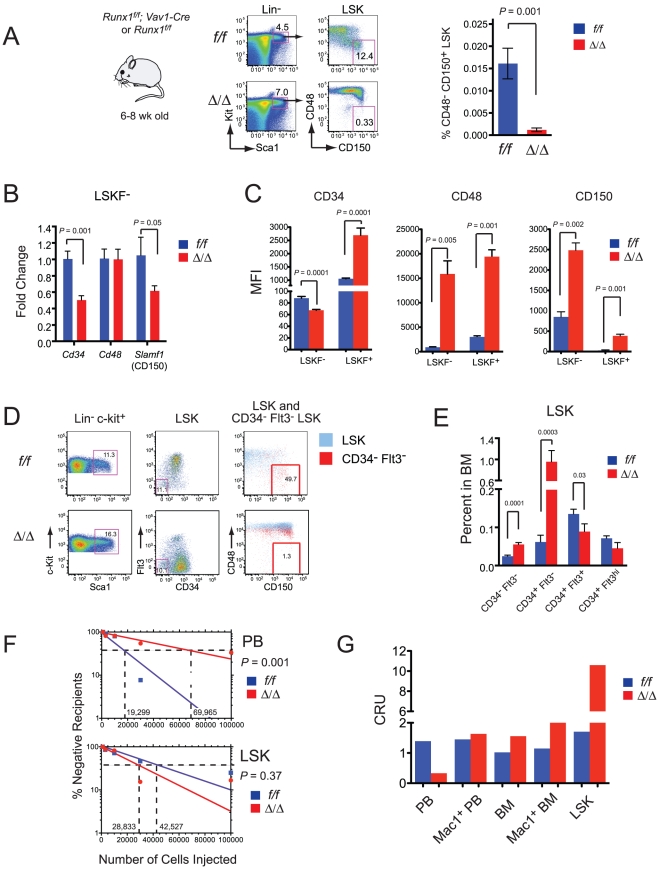
LT-HSC marker expression is altered on Runx1 deficient HSCs. A. Percentage of adult bone marrow LSK cells expressing SLAM markers. The gates were drawn based on fluorescence-minus-one controls using *Runx1^f/f^* (*f/f*) and *Runx1^f/f^;Vav1-Cre* (Δ/Δ) cells. The bar graph on the right represents the percentage of CD48^-^ CD150^+^ LSK cells in total bone marrow, averaged from 6-7 mice. Error bars indicate SEM. B. Real time PCR for mRNA encoding LT-HSC markers in donor derived LSKF^-^ cells sorted from recipients of *Runx1^f/f^* and *Runx1^f/f^; Mx1-Cre* bone marrow. n = 4–6 mice of each genotype. Error bars represent SEM. See Figure 1B for gating strategy. C. Mean fluorescence intensity of CD34, CD48, and CD150 on LSKF^-^ and LSKF^+^ cells (n = 6; error bars  =  SEM. D. Simultaneous analysis of all four LT-HSC markers on *Runx1^f/f^* and *Runx1^f/f^;Vav1-Cre* LSK cells. The LSK population (blue) and CD34^-^ Flt3^-^ LSK population (red) in the middle plots are analyzed for CD48 and CD150 expression in the right hand plots. Note the upward shift in CD48 levels, and rightward shift of C150 on Runx1 deficient HSCs. E. The percentage of phenotypic LT-HSCs and ST-HSCs in the bone marrow of 6-8 week old *Runx1^f/f^* and *Runx1^f/f^; Vav1-Cre* mice determined using CD34 and Flt3 markers. Data are averaged from 6 mice, error bars indicate 95% CI. F. Competitive limit dilution transplant. Bone marrow from 6-8 week old *Runx1^f/f^* or *Runx1^f/f^;Vav1-Cre* mice was injected along with 2×10^5^ Ly5.1^+^ adult bone marrow cells into irradiated Ly5.1/5.2 recipients. Contribution of Ly5.2^+^ cells to peripheral blood (top graph) and bone marrow LSK cells (bottom graph) were assessed. Recipients with ≥1% donor-derived cells at ≥16 weeks were deemed reconstituted. Data are compiled from three different experiments with a total of 5 to 13 mice per data point. G. Repopulating units (RU) determined by contribution to different blood cell populations. Competitor cells (2×10^5^, Ly5.1) were transplanted with either 1×10^5^ *Runx1^f/f^* or 3×10^5^ *Runx1^f/f^; Vav1-Cre* bone marrow cells. RUs were calculated according to Harrison *et al*. [68].

Adult *Runx1^f/f^;Vav1-Cre* bone marrow had a very different cell surface phenotype, i.e. a severe depletion of CD48^-^ CD150^+^ LSK cells (Figure 4A), similar to what was reported by Schindler *et al*. [20] when they deleted Runx1 using Mx1-Cre. The depletion of CD48^-^ CD150^+^ LSK cells was pronounced by postnatal week 1 (not shown), and therefore did not correlate with the transition from the fetal to adult HSC phenotype that takes place between 3 and 4 weeks after birth [34]. The percentage of CD48^-^ CD150^+^ LSK cells in the fetal liver did not change in *Runx1^f/f^;Vav1-Cre* mice up through 18.5 dpc (not shown), thus the loss of SLAM-marked phenotypic LT-HSCs was only apparent in the bone marrow. However, the overall increase in mean fluorescence intensity of CD48 on bone marrow LSK cells (Figure 4A) prompted us to examine the expression of commonly used LT-HSC markers on Runx1 deficient cells. The level of CD34 mRNA was lower in Runx1 deficient LSKF^-^ cells (Figure 4B), and the mean fluorescence intensity of cell surface CD34 was moderately decreased (Figure 4C). CD48 mRNA levels were not significantly changed (Figure 4B), but cell surface CD48 was markedly elevated on LSKF^-^ (17.3 fold), and on LSKF^+^ cells (6.4 fold) (Figure 4C). CD150 levels were also elevated on Runx1 deficient LSKF^-^ (3 fold) and LSKF^+^ cells (14.1 fold), despite the fact that CD150 mRNA levels were decreased (Figure 4B,C). Co-staining for all four markers revealed that the phenotypic LT-HSC population defined using CD34 and Flt3 (and LSK cells in general) was shifted upwards on the CD48 axis, and as a result the majority of CD34^-^ Flt3^-^ LSK cells did not fall within the CD48^-^ gate (Figure 4D). Thus several commonly used LT-HSC markers are dysregulated on Runx1 deficient HSCs, and may be unreliable for the definition of phenotypic HSCs in the adult bone marrow.

We used CD34 in combination with Flt3 to re-examine the composition of LSK cells in the bone marrow of 6–8 week old *Runx1^f/f^;Vav1-Cre* mice. In contrast to the marked decrease in phenotypic LT-HSCs observed using SLAM markers, we found a 2 fold increase in the percentage of CD34^-^ Flt3^-^ LSK cells, and an increase in phenotypic ST-HSCs (CD34^+^ Flt3^-^ LSK), as shown previously [18] (Figure 4E). The percentage of Flt3^+^ LSK cells was lower, in contrast to what was observed upon deletion with Mx1-Cre (Figure 1B).

We used limit dilution transplants to determine which, if any, phenotypic markers correlated with functional outcome. Since Runx1 deletion impairs lymphoid development, while it moderately expands LSK cells and myeloid progenitors in the bone marrow [15], [16], we assessed contribution in several ways. The frequency of LT-HSCs was 3.6 fold lower in young adult (6–8 week old) *Runx1^f/f^;Vav1-Cre* mice, similar to what we observed in the fetal liver, when donor contribution to total nucleated peripheral blood was analyzed (Figure 4F and Table 2). When contribution to myeloid lineage (Mac1^+^) cells in peripheral blood was scored, there was a fairly comparable (2.7 fold) decrease in LT-HSC frequency. However, there was no difference in the frequency of *Runx1^f/f^;Vav1-Cre* versus *Runx1^f/f^* LT-HSCs when contribution to whole bone marrow, bone marrow Mac1^+^ cells, or LSK cells were scored (Figure 4F and Table 2). These discrepancies are caused by differences in the contribution of *Runx1^f/f^;Vav1-Cre* cells to various hematopoietic populations. Calculation of repopulating units (RU) based on contribution to cells in bone marrow or peripheral blood showed that *Runx1^f/f^* LT-HSCs contributed equivalently to total nucleated peripheral blood cells, Mac1^+^ peripheral blood cells, total bone marrow, Mac1^+^ bone marrow cells, and LSK cells (Figure 4G). *Runx1^f/f^;Vav1-Cre* LT-HSCs, on the other hand, contributed unevenly to these populations, with the most dramatic difference in the contribution to total peripheral blood (calculated as 0.3 RU) versus bone marrow LSK cells (10.6 RU). Thus, using a fairly typical cutoff of ≥1% donor derived cells, a small number of recipients scored positive based on contribution to one lineage and negative based on contribution to another. In summary, it appears that the number of phenotypic and functional LT-HSCs in *Runx1^f/f^;Vav1-Cre* mice varies depending on the markers used, and the criteria used to count them. This may explain why independent investigators have come to different conclusions regarding the consequences of Runx1 deletion in the LT-HSC population. Incorporating all of our findings we conclude that there is a moderate, 2–4 fold decrease in the number of Runx1 deficient LT-HSCs in young adult bone marrow if contribution to peripheral blood is assessed, and no difference if contribution to bone marrow is scored. No markers accurately predicted the difference in LT-HSC frequencies in *Runx1^f/f^* versus *Runx1^f/f^;Vav1-Cre* bone marrow. The markers that best approximated functional activity in bone marrow LT-HSCs were CD34^-^ Flt3^-^ LSK, and these were used in all subsequent analyses.

**Table 2:** LT-HSC frequencies in adult bone marrow determined by contribution to different lineages.

Genotype	Donor contribution	Frequency	Upper range (95% CI)	Lower range (95% CI)	Relative frequency compared to *Runx1^f/f^*	*P* value
*Runx1^f/f^*	PB (nucleated cells)	1∶19,299	1∶11,607	1∶32,088		
*Runx1^f/f^*	PB Mac1^+^ cells	1∶21,992	1∶12,661	1∶38,202		
*Runx1^f/f^*	BM (nucleated cells)	1∶26,288	1∶15,266	1∶45,268		
*Runx1^f/f^*	BM Mac1^+^ cells	1∶24,095	1∶13,877	1∶41,838		
*Runx1^f/f^*	BM LSK cells	1∶37,769	1∶21,018	1∶67,869		
*Runx1^f/f^*;*Vav1-Cre*	PB (nucleated cells)	1∶69,965	1∶39,351	1∶12,4396	0.28	0.001
*Runx1^f/f^*;*Vav1-Cre*	PB Mac1^+^ cells	1∶58,961	1∶33,389	1∶104,120	0.37	0.01
*Runx1^f/f^*;*Vav1-Cre*	BM (nucleated cells)	1∶26,120	1∶15,579	1∶43,791	0.20	0.49
*Runx1^f/f^*;*Vav1-Cre*	BM Mac1^+^ cells	1∶26,118	1∶15,581	1∶43,783	1.71	0.42
*Runx1^f/f^*;*Vav1-Cre*	BM LSK cells	1∶28,833	1∶17,089	1∶48,649	1.47	0.25

### Runx1 loss slows HSC proliferation and reduces apoptosis

**Figure 5:**
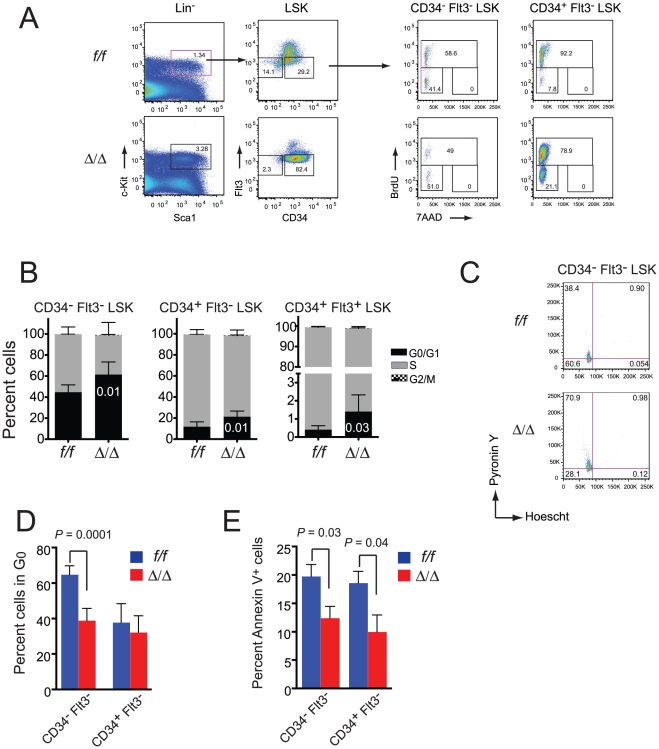
Alterations in cell cycle and apoptosis in Runx1 deficient adult HSCs. A. Representative scatter plots for BrdU cell cycle analysis. Mice were exposed to BrdU for three days prior to analysis. CD34^-^ Flt3^-^ LSK and CD34^+^ Flt3^-^ LSK cells were analyzed for BrdU and DNA content in plots on the right. B. Summary of data from a total of 9 *Runx1^f/f^* and *Runx1^f/f^;Vav1-Cre* mice. Error bars indicate 95% CI. *P* values for differences between wild type and mutant cells in G_0_/G_1_ are indicated in the bars. C. *Runx1^f/f^* and *Runx1^f/f^;Vav1-Cre* CD34^-^ Flt3^-^ LSK cells were sorted from bone marrow by multicolor FACS, stained with Hoechst 33342 and Pyronin Y, and analyzed by flow cytometry. Scatter plots show representative cell cycle distributions. D. Summary of quiescence analysis (n = 6 to 8). Error bars represent 95% CI. E. Annexin V staining of *Runx1^f/f^* and *Runx1^f/f^;Vav1-Cre* CD34^-^ Flt3^-^ LSK and CD34^+^ Flt3^-^ LSK bone marrow cells (5-7 mice). Error bars represent SEM.

We examined various phenotypic parameters of Runx1 deficient HSCs to gain insights into the pre-leukemic state. BrdU labeling revealed that the Runx1 deficient LT-HSCs, ST-HSCs, and MPPs in the adult bone marrow cycled more slowly than their wild type counterparts, with more cells remaining in G_0_/G_1_ (Figure 5A,B). There were fewer quiescent CD34^-^ Flt3^-^ LSK cells in the *Runx1^f/f^;Vav1-Cre* marrow (Figure 5C,D), whereas quiescence in the ST-HSC population (CD34^+^ Flt3^-^ LSK) was not altered. Thus subtle alterations in cell cycle properties result in somewhat more LT-HSCs exiting quiescence, but all LSK cells progressing more slowly through cell cycle and accumulating in G_1_. There were fewer Annexin V^+^ CD34^-^ Flt3^-^ LSK and CD34^+^ Flt3^-^ LSK cells (Figure 5E), suggesting that Runx1 positively regulates apoptosis in HSCs, as noted previously [17]. Thus LSK expansion may be caused by a decreased rate of apoptosis, but not by increased proliferation.

**Figure 6:**
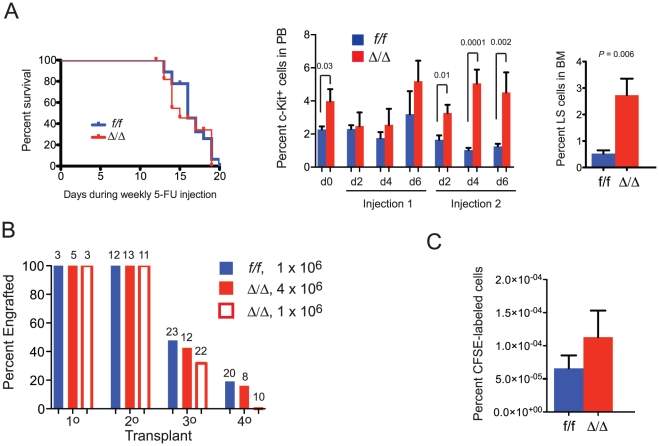
Runx1 deficient HSCs are not exhausted by proliferative stress. A. Kaplan-Meier survival curve of mice following weekly injections of 5-FU (on days 1, 7, 14) is on the left. *Runx1^f/f^*, n = 10; *Runx1^f/f^;Vav1-Cre*, n = 9. The middle graph represents the percentage of c-Kit^+^ cells in peripheral blood before 5-FU injection (d0), and various days following weekly injection (6-12 mice per data point). Error bars are SEM. Right hand graph is percentage of LS cells in the bone marrow 7 days after the second 5-FU injection. *Runx1^f/f^*, n = 7. *Runx1^f/f^;Vav1-Cre*, n = 8. B. Serial transplantation of bone marrow cells. Numbers above bars indicate the number of transplant recipients, not including those that died within two weeks from radiation toxicity. The genotypes and doses represent the bone marrow cells used to engraft primary recipients. 2×10^6^ cells from primary recipients were transplanted into secondary recipients, and the process was repeated twice more. C. Homing assay. CD34^-^ Flt3^-^ LSK cells were isolated by FACS, labeled with CFSE, and 1000 cells transplanted into 4-5 recipients along with 2 x10^5^ unlabeled carrier cells. Bone marrow cells (>10^7^) were analyzed 16 hours later by FACS. Shown is the percentage of live CFSE^+^ Ter119^-^ cells. Data are representative of two experiments.

We examined the effect of Runx1 deficiency on the response to myelotoxic stress. There was no difference in the survival of *Runx1^f/f^* and *Runx1^f/f^;Vav1-Cre* mice following weekly injections with 5-fluorouracil (5-FU), which kills cycling cells (Figure 6A). Immature c-Kit^+^ cells in the peripheral blood of *Runx1^f/f^;Vav1-Cre* mice were elevated before 5-FU treatment, and after the second 5-FU injection (Figure 6A). The percentage of *Runx1^f/f^;Vav1-Cre* LS cells in the bone marrow (cell surface c-Kit in bone marrow is decreased by 5-FU and could not be used as a marker [35]) was 5-fold higher than that of *Runx1^f/f^* LS cells following two rounds of 5-FU injections (Figure 6A). Therefore Runx1 deficiency does not sensitize mice or HSCs/progenitors to myelotoxic stress.

We also examined the effect of forced proliferation of LT-HSCs through serial transplantation. Since the frequency of Runx1 deficient LT-HSCs in the bone marrow is either unaltered or lower by approximately 3–4 fold (Table 2), we transplanted primary recipients with two different doses of *Runx1^f/f^;Vav1-Cre* bone marrow cells, either equal to the number of control *Runx1^f/f^* cells (1×10^6^), or four fold more (4×10^6^), all without competitors. Bone marrow from each primary recipient (2×10^6^ cells) was then serially transplanted three times, and donor contribution to Mac1^+^ peripheral blood cells assessed (Figure 6B). When an initial dose of 1×10^6^ bone marrow cells was used, no recipients of *Runx1^f/f^;Vav1-Cre* cells were engrafted in the fourth round, compared to 19% of mice transplanted with *Runx1^f/f^* cells. However, the fraction of reconstituted 3^0^ and 4^0^ recipients was the same when starting doses of 1×10^6^ *Runx1^f/f^* and 4×10^6^ *Runx1^f/f^;Vav1-Cre* bone marrow cells, which contain almost equivalent numbers of LT-HSCs, were transplanted (Figure 6C). Therefore, although the frequency of LT-HSCs was 3-4 fold lower in the original *Runx1^f/f^;Vav1-Cre* bone marrow, the LT-HSCs were normal with respect to the efficiency with which they engrafted and expanded in the subsequent transplant recipients, and in their self-renewal. Consistent with the similar engraftment efficiency, there was no significant difference in the ability of CFSE-labeled CD34^-^ Flt3^-^ LSK cells from *Runx1^f/f^;Vav1-Cre* mice to home to the bone marrow (Figure 6C).

### Genes and proteins dysregulated in Runx1 deficient HSCs

**Figure 7:**
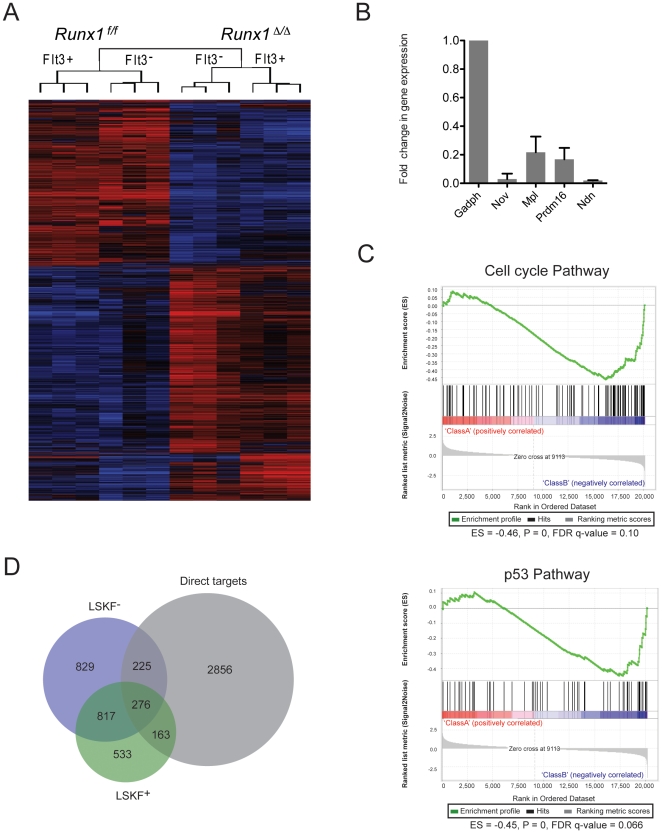
Microarray analysis of genes mis-expressed in Runx1 deficient HSCs. A. Heat plot of 4707 probe sets, representing 3820 genes, differentially expressed between LSKF^-^ and LSKF^+^ cells isolated from mice transplanted with *Runx1^f/f^* and *Runx1^f/f^; Mx1-Cre* bone marrow, with a significance threshold of *P = *0.005. Each column shows gene expression from donor cells from an independent transplant recipient. Red, > than the average, blue, < than the average. B. Quantitative real-time PCR (qRT-PCR) performed on several genes to independently validate the microarray data. Data shown are the mean fold change in expression (± SEM), normalized to *Gapdh* expression, between *Runx1^Δ/Δ^* (n = 3) and *Runx1^f/f^* (n = 3) LSKF^-^ samples. C. GSEA profiles illustrating the correlation between genes negatively regulated by Runx1 in LSK cells, and genes sets of cell cycle and p53 pathways. Enrichment score, p values and FDR q-values are shown under the enrichment plots. Number of permutations was set to 1000. D. Venn diagram illustrating overlap between dysregulated genes in Runx1 deficient LSKF^-^ and LSKF^+^ cells, and genes occupied by Runx1 in the HPC-7 line [44]. For list of genes see Table S4.

We examined changes in the global gene expression profiles to gain insights into the molecular mechanisms by which Runx1 loss creates preleukemic HSCs. Bone marrow chimeras stably reconstituted by donor *Runx1^+/+^; Mx1-Cre^+/+^* or *Runx1^f/f^; Mx1-Cre* marrow were injected with pIpC to induce Cre, and donor-derived LSK, LSKF^-^, and LSKF^+^ cells from individual recipients purified by cell sorting. Total RNA was harvested from each sample and analyzed using Affymetrix Mouse Genome 430 2.0 GeneChip arrays. Using a significance threshold of 0.005, we identified 902 unique genes that were differentially expressed ≥ 1.5 fold between *Runx1^+/+^* and Runx1-deficient (*Runx1^Δ/Δ^*) LSK cells (Table S1), 2147 differentially expressed genes in LSKF^-^ cells (Figure 7A and Table S2), and 1789 genes in LSKF^+^ cells (Figure 7A and Table S3). Some of the most highly under-expressed genes in LSKF^-^ cells included *Nov*, *Mpl*, and *Prdm16*, all of which encode positive regulators of HSC self-renewal or engraftment (Figure 7B) [36], [37], [38], [39]. One of the most highly under-expressed genes was *Ndn*, which was previously implicated in regulating HSC quiescence [40], although later studies found no role for *Ndn* in maintaining steady state hematopoiesis or protecting against stem cell exhaustion [41]. Many genes implicated in controlling HSC quiescence, or the distribution between G_0_/G_1_ and S/G_2_/M, including *Gfi1, Pten*, *Foxo* family genes, *Elf4, Cdkn2c, Hoxb4, Myc*, *Mll1*, *Junb*, *Cdkn2a,* and *Tp53* [40], [42], [43] were not affected by Runx1 deficiency, although Gene Set Enrichment Analysis (GSEA) identified both the cell cycle and p53 pathways as being significantly dysregulated (Figure 7C). Of the 2147 and 1789 genes whose expression was dysregulated in either or LSKF^-^ or LSKF^+^ cells, respectively, 664 contained a consensus site that was occupied by Runx1 in a human hematopoietic progenitor cell line [44] (Figure 7D and Table S4), thus the majority are likely to be indirect Runx1 targets. We note, though, that the gene expression analysis may be compromised by alterations in the expression of markers used to isolate the populations in question.

## Discussion

We examined the cell-intrinsic properties of Runx1 deficient HSCs using both phenotypic and functional assays. One of our goals was to attempt to reconcile the contradictory reports in the literature regarding the effects of Runx1 loss on phenotypic and functional HSCs. We discovered that one of the inherent difficulties in studying Runx1 deficient HSCs, which may have contributed to some of the discrepancies in the literature, is the uncertainty regarding phenotypic markers. Specifically, we found that markers commonly used to subdivide the LSK population (CD34/Flt3 versus CD48/CD150) yielded different phenotypic LT-HSC frequencies. This may be caused, in part, by direct regulation by Runx1 of genes encoding one or more of these markers, as has been noted previously with other antigens commonly used to define phenotypic blood cell populations in Runx1 deficient animals. The most well known example of this is the derepression of CD4 expression on thymocytes. *Cd4* contains an extensively characterized silencer element that is regulated by Runx1, and deletion of either Runx1 or the silencer causes inappropriate CD4 expression in double negative (CD4^-^ CD8^-^) thymocytes [45]. A recent ChIP-Seq analysis of the HPC-7 hematopoietic progenitor cell line showed that all but one gene encoding commonly used HSC markers (*Kit, Flt3, Cd34, Cd48, Slamf1/CD150*) were occupied by Runx1 and thus are direct Runx1 targets [44]. However not all marker genes occupied by Runx1 appear to be regulated at the level of mRNA production or stability. *Cd48* mRNA, for example, is present in normal amounts, but cell surface CD48 is strikingly elevated on Runx1 deficient LSK cells. The mechanistic basis for CD48 dysregulation is unclear. One possibility is that Runx1 indirectly regulates CD48 translation through a microRNA. However, TargetScan identified no microRNA target sites in the *Cd48* 3′UTR, and while PicTar identified a site for mmu-miR-31, the expression of mmu-miR-31 is upregulated rather than downregulated in Runx1 deficient HSCs (not shown). The pronounced elevation of cell surface CD48 levels is the first example of a dysregulated SLAM marker of which we’re aware.

Runx1 deficiency blocks lymphocyte development, while at the same time expands LSK cells and granulocyte progenitors in the bone marrow [14], [15], [16], [17], [18]. These features complicate the evaluation of functional LT-HSCs, the methods for which generally employ a cutoff value for positive engraftment. Thus, we obtained slightly different LT-HSC frequencies depending on the lineage we assessed. LT-HSC frequencies were 3–4 fold lower in *Runx1^f/f^;Vav1-Cre* bone marrow when contribution to peripheral blood was scored, but not significantly different from those in *Runx1^f/f^* mice when contribution to bone marrow was assessed. The difference in LT-HSC frequencies calculated from the peripheral blood and bone marrow could be caused by impaired output of Runx1 deficient HSCs to differentiated progeny in the peripheral blood, from selective expansion of HSCs and progenitors in the bone marrow, or both. Ichikawa *et al.* [16] previously reported that LT-HSC frequencies were elevated 10 fold when Runx1 was deleted in all bone marrow cells with Mx1-Cre, which they assessed by PCR analysis for Y-chromosome positive donor-derived cells in the bone marrow of female recipients. Although we observed no difference in the frequency of wild type and Runx1 deficient HSCs when assessing bone marrow engraftment, the two experiments are difficult to compare since they used different deletion strategies, and the sensitivity and selectivity of the techniques used to score engraftment were also dissimilar. Jacob *et al.* [18], on the other hand, reported a 2.7 fold decrease in the frequency of LT-HSCs between aged (40 week old) *Runx1^f/f^;Mx1-Cre* mice and *Runx1^f/f^* mice, and concluded that Runx1 deficiency caused an age-dependent HSC exhaustion. However, Jacob *et al.* [18] did not compare the frequency of functional LT-HSCs in young adult mice. Since we observed similar decreases in LT-HSC frequencies in the fetal liver (4.0 fold) and in young adult bone marrow (3.6 fold) when we assessed contribution to peripheral blood, we do not think the 2.7 decrease observed by Jacob *et al.* in old mice is due to exhaustion. This interpretation is supported by 5-FU and serial transplant experiments, which uncovered no disadvantage conferred by Runx1 deficiency.

Biallelic or monoallelic mutations in *RUNX1* are found in myeloid malignancies, and in many cases are thought to be initiating mutations that generate a pre-leukemic HSC. We endeavored to define the properties of Runx1 deficient HSCs that contribute to this pre-leukemic state. A potent pre-leukemic HSC should be retained in the bone marrow for many years, clonally expand, and exhibit an elevated level of genetic or epigenetic instability. Runx1 deficient HSCs have properties consistent with at least two of these features. First, and most importantly, Runx1 deficient HSCs do not undergo exhaustion and therefore perdure. We note that loss of function mutations that negatively impact HSC engraftment or activity (*Pten, Gfi1, Foxo1,3,4, Bmi1, Moz, Mll1*) [46], [47], [48], [49], [50], [51], [52], [53], [54] are either uncommon or not observed in AML, perhaps because HSCs that acquire these mutations as a first hit have a competitive disadvantage in the bone marrow or are rapidly lost.

The perdurance of Runx1 deficient HSCs may be conferred, in part, by their slow cycling behavior. Runx1 deficient HSCs contain a significantly higher percentage of cells in G_1_, thus Runx1 normally functions to promote cell cycle progression. This is an evolutionarily conserved property of Runx1 in stem cell populations. For example, the *C. elegans* homologue RNT-1 promotes the proliferation of lateral hypodermic stem cells (seam cells), and sea urchin Runx (*SpRunt-1*) is necessary for the proliferation of late stage blastula cells [55], [56]. In mammals, Runx1 sustains proliferation and prevents the differentiation of transient amplifying progenitors and precursors of olfactory sensory neurons, and promotes proliferation of hair follicle stem cells and keratinocytes [57], [58]. However Runx1 can also repress proliferation. For example, overexpression of Runx1 in hematopoietic progenitors cultured in vitro increased the proportion of cells in G_1_ [59], and ectopic expression of Runx1 in primary mouse and human fibroblasts induced a senescence-like growth arrest with an accumulation of cells in G_1_ [60], [61], [62]. Whether Runx1 promotes or represses proliferation appears to be cell context dependent, and the rules governing which activity will predominant are poorly understood.

The slow replication of Runx1 deficient HSCs could protect them from genotoxic stress, leading to selective expansion. It has long been known that cultured cells in early G_1_ are relatively radioresistant [63], [64]. It was recently shown that inducing a pharmacological G_1_ arrest in HSCs and progenitors mitigated hematological toxicity and mortality following total body irradiation [65]. Delays in cell cycle progression in hematopoietic stem and progenitor cells are not unique to Runx1 loss, and were also observed in cells expressing the dominant inhibitors AML1-ETO and CBFß-SMMHC [28], [29], [31], [66]. The defective differentiation of Runx1 deficient cells along the lymphoid and megakaryocytic pathways would, of course, contribute to the clinical features of the full-blown disease, such as the multilineage dysplasia in MDS, or the poorly differentiated blast cells in AML.

In summary, we have attempted to resolve controversies in the literature regarding the effect of Runx1 loss on both phenotypic and functional HSCs. We conclude that the negative impact of Runx1 loss on the frequency and self-renewal of functional LT-HSCs is only modest or absent. Runx1 deficient HSCs replicate slowly and persist in the bone marrow, providing a pool of preleukemic HSCs poised to acquire secondary mutations that would promote their growth and progression to MDS or AML.

## Supporting Information

Table S1 **Gene expression changes in LSK cells.** Listed are changes >1.5 fold, with a significance threshold of 0.005. The data correspond to 1026 probesets and 902 unique genes.
(XLS)

Table S2 **Gene expression changes in LSKF^-^ cells.** Listed are changes >1.5 fold, with a significance threshold of 0.005. The data correspond to 2526 probesets and 2147 unique genes.
(XLS)

Table S3 **Gene expression changes in LSKF^+^ cells.** Listed are changes >1.5 fold, with a significance threshold of 0.005. The data correspond to 2048 probesets and 1789 unique genes.
(XLS)

Table S4 **Overlap of differentially expressed genes, and genes occupied by Runx1 in HPC-7 cells according to Wilson et al.** [**44**] **.** Column A lists genes occupied by Runx1 [44]. Columns C, E, and G are genes differentially expressed in Runx1 deficient LSKF^-^, LSKF^+^ or in both LSKF^-^ and LSKF^+^ cells. Columns I, K, and M list overlap between differentially genes and occupied genes in each cell population.
(XLS)
